# Self-Calibration of Angular Position Sensors by Signal Flow Networks

**DOI:** 10.3390/s18082513

**Published:** 2018-08-01

**Authors:** Zhenyi Gao, Bin Zhou, Bo Hou, Chao Li, Qi Wei, Rong Zhang

**Affiliations:** 1 Engineering Research Center for Navigation Technology, Department of Precision Instrument, Tsinghua University, Beijing 100084, China; gaozy17@mails.tsinghua.edu.cn (Z.G.); houb15@mails.tsinghua.edu.cn (B.H.); cli16@mails.tsinghua.edu.cn (C.L.); 2 Department of Electronic Engineering, Tsinghua University, Beijing 100084, China

**Keywords:** self-calibration, signal flow network, signal processing, angular position sensor

## Abstract

Angle position sensors (APSs) usually require initial calibration to improve their accuracy. This article introduces a novel offline self-calibration scheme in which a signal flow network is employed to reduce the amplitude errors, direct-current (DC) offsets, and phase shift without requiring extra calibration instruments. In this approach, a signal flow network is firstly constructed to overcome the parametric coupling caused by the linearization model and to ensure the independence of the parameters. The model parameters are stored in the nodes of the network, and the intermediate variables are input into the optimization pipeline to overcome the local optimization problem. A deep learning algorithm is also used to improve the accuracy and speed of convergence to a global optimal solution. The results of simulations show that the proposed method can achieve a high identification accuracy with a relative parameter identification error less than 0.001‰. The practical effects were also verified by implementing the developed technique in a capacitive APS, and the experimental results demonstrate that the sensor error after signal calibration could be reduced to only 6.98%.

## 1. Introduction

Obtaining accurate angle information is crucial for many control systems [1,2,3]. Therefore, angle position sensors (APSs) are widely used in the aerospace and automotive industries, navigation, and other fields. Ideally, sensors including resolvers [4,5] and capacitive angular position sensors (CAPSs) [6,7] modulate the angle signals and output sets of orthogonal sine and cosine signals. In practical applications, due to processing error, installation error, circuit mismatch, etc., output signals may be disturbed, resulting in unexpected errors such as amplitude deviations, direct-current (DC) offsets, and phase offsets. To obtain an accurate angle signal, it is necessary to identify the signal model parameters and calibrate the output signals. The calibration yields an accurate signal based on the identified parameters.

To improve the angle signal accuracy, many research methods have been proposed in recent years. Le et al. [8] presented a quadrature digital phase-locked loop method and used interpolation to improve the accuracy of the position information. Kim et al. [1] obtained additional reference signals for calibration and demodulation through a D-Q transform of the motor. Both calibration methods attempt to suppress the error sources in the signal model and require additional hardware costs. Hoang et al. [9] proposed establishing a look-up table for compensation. This method requires additional hardware storage space and does not provide accurate parameter values. Dhar et al. [10] used an artificial neural network to compensate for angle errors, and Tan et al. [11] employed a radial basis function neural network for angle calibration. These methods do not identify the parameter values either. All of the above techniques require obtaining an accurate reference input signal, making it difficult to perform parameter self-calibration, which limits the use of these methods in practical applications.

Selecting an appropriate optimization algorithm is another method of parameter identification and calibration. The optimization technique is essentially based on the least squares method. An optimal solution extraction process for parameters was first introduced in [12]. On this basis, gradient-based identification algorithms with model linearization have also been proposed [9,13]. These methods permit self-calibration. However, new parameters are coupled to the original independent parameters after linearization. The parameters do not maintain independence and may result in large identification errors. In addition, the traditional gradient descent method does not have the ability to escape local optima. According to the method described by Hou [14] for parameter identification of sinusoidal signals, self-calibration methods based on observers have been presented. Zhang et al. [15] proposed an automatic calibration scheme based on a state observer, and Wu et al. [16] introduced a two-step identification improvement scheme for the above method. This kind of scheme entails more stringent output signal requirements. The output signal must be continuous and second-order differentiable. For applications in which the angle measurement range is less than 360° and the output signal is not smooth, its usability is limited. A method based on Fourier analysis has also been introduced for self-calibration [17,18]. This technique is effective and can be used for online calibration, but the influences harmonic contents are ignored, and there is no discussion of this approach in the literature.

In this article, a novel offline self-calibration scheme is introduced. The new method is data driven, and there are no special requirements on the data, such as input signal continuity. This method also does not impose linearization on the model. By establishing optimization operation pipelines, the scheme has the ability to guarantee that the iterative learning process moves toward the global optimum. In this paper, this approach is called calibration based on a signal flow network (CSFN) method. In this technique, the relationship between the output signal of the sensors and the angle signal to be measured is converted into a signal flow network structure. According to the CSFN, the sensor output is used as a new input, the relationship between two sensor outputs is transferred to the network structure. The parameters are set as the nodes of the network. The network output is related to the parameters and the network input signal. The CSFN model was designed in a deep learning framework [19], using an optimization algorithm to conduct the identification process, and experimental data were collected for offline identification and calibration. Simulations and experiments demonstrated that this method can provide effective calibration.

The remainder of this paper is organized as follows: in Section 2, the signal model and error model are discussed. Section 3 presents the signal flow network construction method and parameter identification scheme. In Section 4, the simulation and experiments conducted to verify the validity of the CSFN method are described. Section 5 and Section 6 respectively provide a discussion of the results and our conclusions.

## 2. Signal Model and Problem Statement

### 2.1. Signal Model

**Figure 1:**
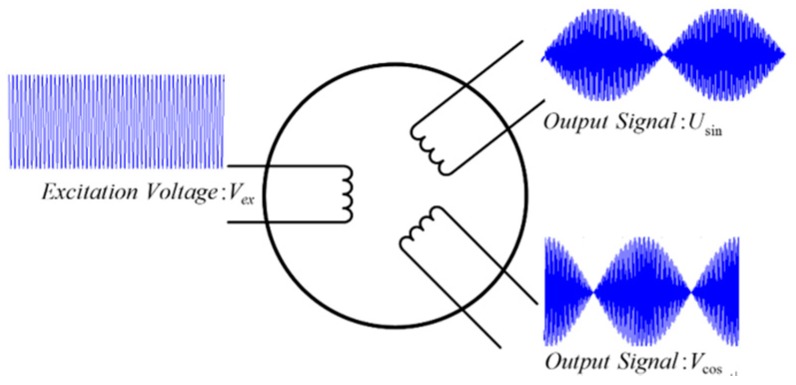
Classic input and output model of an APS.

Figure 1 shows the classic model of an APS, including resolvers and a CAPS. Under the influence of an excitation voltage, the sensor outputs two signals, denoted as $U_{sin}$ and $V_{cos}$. The two output signals of the sensor can be expressed as [6,20]:

(1) $$\begin{array}{c} U_{sin}=k\cdot E\cdot \cos\left(\omega \cdot t\right)\cdot \sin\theta \\ V_{cos}=k\cdot E\cdot \cos\left(\omega \cdot t\right)\cdot \cos\theta . \end{array}$$

In Equation (1), k is the gain coefficient of the sensor, ω is the frequency of the excitation voltage, E is the amplitude of the excitation voltage, and θ is the angle to be measured.

Ideally, the sensor output signals are envelope-detected [6] and can be expressed as follows:

(2) U=sinθ, V=cosθ.

**Figure 2:**
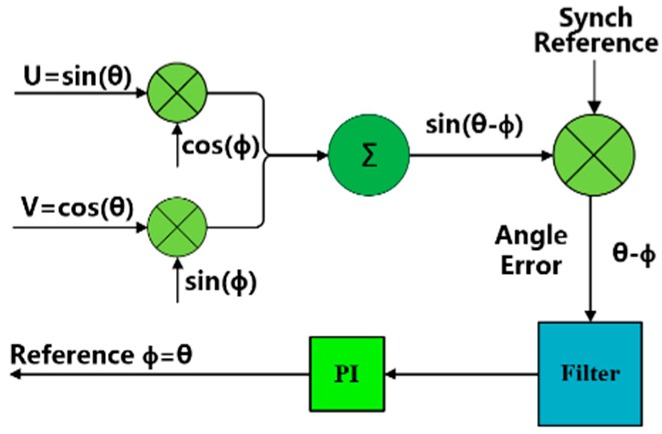
Block diagram of angle demodulation.

Then, the two output signals are sent to the digital demodulation circuit shown in Figure 2. The angle to be measured can be obtained using a proportional-integral controller module [6].

### 2.2. Problem Statement

In practical applications, the imbalance of the mechanical and circuit structure of the sensor will cause amplitude deviation. Zero offset of the sensor can cause DC offset of the output signals. There also exists a phase shift between the output signals [21]. Thus, the actual forms of the output signals are:

(3) $$\begin{array}{c} U=a_{1}\cdot \sin\theta +b_{1}\\ V=a_{2}\cdot \cos\left(\theta +\beta \right)+b_{2}. \end{array}$$

In Equation (3), $a_{1}$ and $a_{2}$ are the real amplitudes, $b_{1}$ and $b_{2}$ are the DC offsets, and β is the non-orthogonal phase shift between the two output signals.

If the above forms of the signals are used to perform angle demodulation, there will be measurement error. Therefore, it is necessary to identify the amplitudes, DC offsets, and phase shift of the output signals to obtain an accurate demodulation angle.

**Figure 3:**
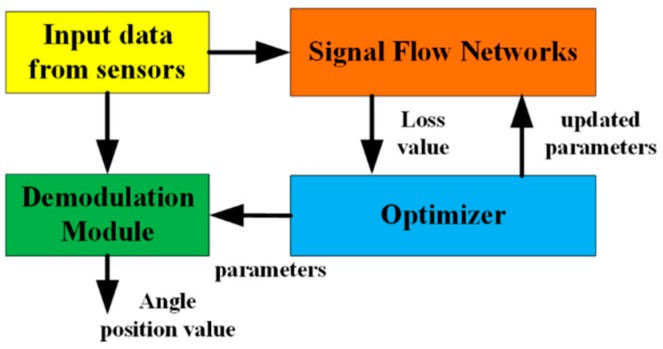
Block diagram of the proposed method.

As shown in Figure 3, the proposed scheme includes three main parts.

Firstly, the CSFN is designed, and the network outputs the loss values associated with the parameters. Secondly, the optimizer uses the loss values from the network to update the parameters. Thirdly, when the loss value converges or is less than a certain preset threshold, the parameters are output. Then, the signal calibration is conducted according to the following formulas:

(4) $$\begin{array}{c} {\hat{U}}_{sin}=\left(U-{\hat{b}}_{1}\right)/{\hat{a}}_{1}=\sin\theta \\ {\hat{V}}_{cos}=V-{\hat{b}}_{2}/{\hat{a}}_{2}\cos\beta +\left(U-{\hat{b}}_{2}\right)\tan\beta =\cos\theta . \end{array}$$

In Equation (4), ${\hat{a}}_{1}$ and ${\hat{a}}_{2}$ are the identified amplitudes, ${\hat{b}}_{1}$ and ${\hat{b}}_{2}$ are the identified DC offsets, and β is the identified phase shift between the two output signals.

## 3. CSFN Design and Parameter Update Principles

**Figure 4:**
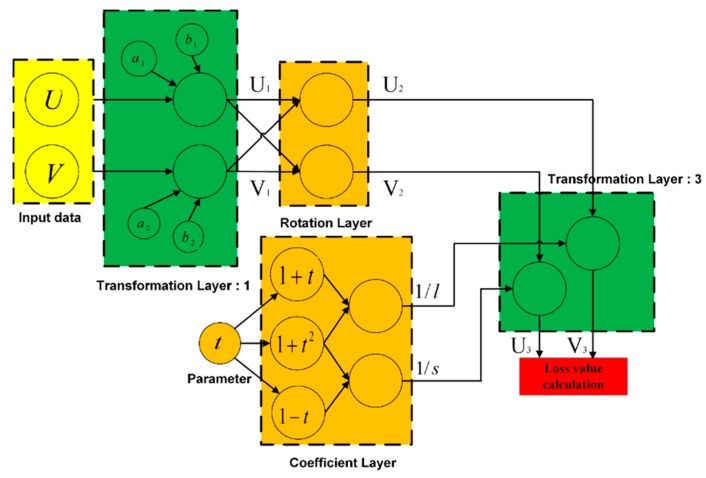
Structure of the signal flow network.

Figure 4 shows the structure of the signal flow network. Since the parameters and reference angle signals are unknown, this method attempts to identify some mappings so that the problem can be solved in the new mapping space. The independence of the parameters is maintained in the mapping process, which is the basis for the optimization analysis. 

The basic working principle of this network is as follows. By performing function transformations, the network outputs a function value that is related to the parameters. The output function value is unrelated to the angle to be measured. This function is called a loss function. During the network training process, the loss function value gradually decreases and the updated parameter values from the back-propagation gradient flow can be obtained.

### 3.1. CSNF Design

The CSNF design is hierarchical. The outputs U and V of the sensor have the form shown in Equation (3). As the network input data, through an affine transformation layer, the outputs $U_{1}$ and $V_{1}$ can be expressed as follows:

(5) $$\begin{array}{c} U_{1}=\left(U-b_{1}\right)/a_{1}=\sin\theta \\ V_{1}=\left(V-b_{2}\right)/a_{2}=\cos(\theta +\beta ). \end{array}$$

In Equation (5), $U_{1}$ and $V_{1}$ are the elliptic parametric equations for θ. The following relationship can be verified:

(6) $$\frac{V_{1}^{2}}{\cos^{2}\beta }+\frac{U_{1}^{2}}{\cos^{2}\beta }+\frac{2\tan\beta }{\cos\beta }\cdot U_{1}\cdot V_{1}=1.$$

**Figure 5:**
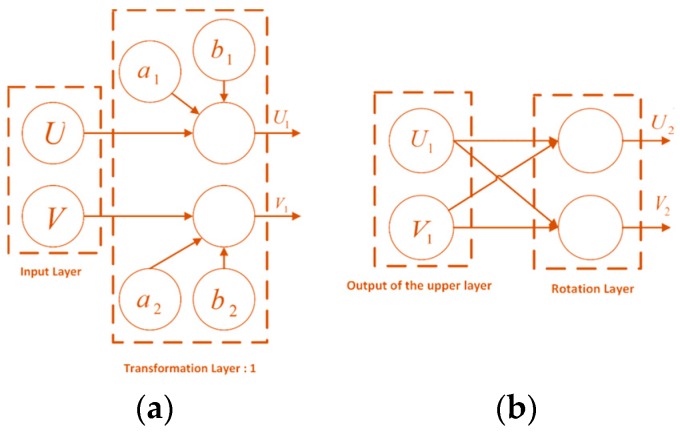
Structures of the transformation layers. (**a**) First layer; (**b**) rotation layer.

The structures of the input layer and first transformation layer are shown in Figure 5a. The circles in the figure indicate the net nodes, and the symbols in the circles represent the structural parameters. The lines between the nodes indicate that there exist function transformations.

The angle between the major axis of the ellipse and the x-axis is 45°. This ellipse can be mapped to a unit circle by performing a rotation transformation and another affine transformation. The rotation matrix is:

(7) $$R=\left(\begin{array}{cc} \sqrt{2}/2 & \sqrt{2}/2\\ -\sqrt{2}/2 & \sqrt{2}/2 \end{array}\right).$$

The output of the rotation transformation layer can be expressed as:

(8) $$\left(\begin{array}{c} V_{2}\\ U_{2} \end{array}\right)=R\left(\begin{array}{c} V_{1}\\ U_{1} \end{array}\right)=\left(\begin{array}{cc} \sqrt{2}/2 & \sqrt{2}/2\\ -\sqrt{2}/2 & \sqrt{2}/2 \end{array}\right)\left(\begin{array}{c} V_{1}\\ U_{1} \end{array}\right)=\frac{\sqrt{2}}{2}\left(\begin{array}{c} V_{1}-U_{1}\\ V_{1}+U_{1} \end{array}\right).$$

Figure 5b depicts the structure of the second transformation layer. By using the outputs of the rotation transformation layer $U_{2}$ and $V_{2}$, outputs $U_{1}$ and $V_{1}$ can be represented as:

(9) $$\begin{array}{c} V_{1}^{2}+U_{1}^{2}=\frac{1}{2}\left(V_{2}^{2}+U_{2}^{2}\right)\\ V_{1}\cdot U_{1}=\frac{1}{2}(-V_{2}^{2}+U_{2}^{2}). \end{array}$$

Then the expressions can be brought plugged into Equation (6) to obtain the following expression relating $U_{2}$ and $V_{2}$:

(10) $$\frac{V_{2}^{2}}{l^{2}}+\frac{U_{2}^{2}}{s^{2}}=1.$$

In practical applications, β has a small numerical value. Assuming that β is less than 90°, the formulas for calculating the major and minor axes are:

(11) $$l=\frac{\cos\beta }{\sqrt{1-\sin\beta }},\;s=\frac{\cos\beta }{\sqrt{1+\sin\beta }}.$$

Equation (10) shows that the relationship between $U_{2}$ and $V_{2}$ takes the form of a standard ellipse equation.

**Figure 6:**
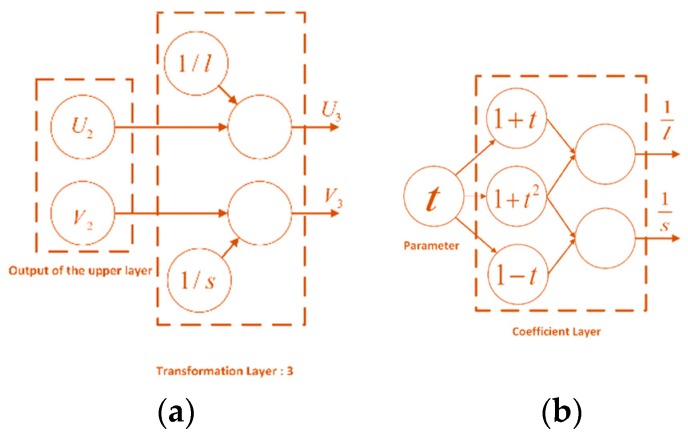
Structures of the transformation layers. (**a**) Third layer; (**b**) coefficient layer.

Above are the coefficient transformation formulas. Figure 6a shows the structure of the third translation layer. After the third affine transformation, the outputs can be expressed as:

(12) $$V_{3}=\frac{V_{2}}{l},\;U_{3}=\frac{U_{2}}{s}.$$

However, the transformation parameters of this layer are related to β and are not independent. In the subsequent back propagation of the gradient, the last stage of the gradient propagation should have independent parameters. Therefore, a coefficient layer is included to convert β. The affine transform coefficients l and s are output by the coefficient layer.

To create the coefficient layer, t=tan(β/2) is defined as the new node. The following relationships between the transform coefficients and node parameter t can be obtained:

(13) $$\begin{array}{c} \sin\beta =\frac{2t}{1+t^{2}},\;\cos\beta =\frac{1-t^{2}}{1+t^{2}}\\ \frac{1}{l}=\frac{\sqrt{1-\sin\beta }}{\cos\beta }=\frac{\sqrt{1+t^{2}}}{1+t}\\ \frac{1}{s}=\frac{\sqrt{1+\sin\beta }}{\cos\beta }=\frac{\sqrt{1+t^{2}}}{1-t}. \end{array}$$

After all of the layers have been constructed, the network output is defined as:

(14) $$Y=\sqrt{U_{3}^{2}+V_{3}^{2}}.$$

The network output value should equal 1. Therefore, the loss function is defined as:

(15) $$L={\left(1-Y\right)}^{2}.$$

All of the designs are layered, which makes gradient back propagation and parameter updating simple. The parameter identification procedure is an optimization process that minimizes the loss function values. The next section introduces the identification method, including the parameter update guidelines and network training methods.

### 3.2. Parameter Identification Process

#### 3.2.1. Parameter Update Formula

Stochastic gradient descent is commonly used in optimization theory, but it is difficult to find a suitable learning rate. In addition, it is easy to obtain a local optimal solution. In contrast, the momentum method [22] can escape local optima. In the review presented in [23], the Adam optimization algorithm [24] was chosen for parameter updating. The Adam optimization algorithm can adjust the learning rate adaptively, and it has a certain ability to avoid local optima. By calculating the first and second moment estimates of the gradient, an independent adaptive learning rate is designed for different parameters. This algorithm has significant advantages over other kinds of random optimization algorithms in practice.

The parameter vector is defined as ψ, and X is defined as the input vector. There exists a mapping function f, so the output loss value y can be expressed as:

(16) y=f(ψ,X).

The gradient formula of the loss function related to the parameters is: $g=\frac{\delta f}{\delta X}$.

The Adam optimization algorithm [24] firstly sets four parameters: α is the step size, $\beta_{1}$ and $\beta_{2}$ are the exponential decay rates for moment estimation, and ε is a constant for numerical stability. The default settings are:

(17) $$\alpha =0.001,\;\beta_{1}=0.9,\;\beta_{2}=0.999,\;\varepsilon =10^{-8}.$$

The calculated gradient value is defined as g. Then, the parameters can be updated according to the following principles:

(18) $$\begin{array}{c} T\leftarrow T+1\\ m_{T}\leftarrow \beta_{1}\cdot m_{T-1}+\left(1-\beta_{1}\right)\cdot g\\ v_{T}\leftarrow \beta_{2}\cdot v_{T-1}+\left(1-\beta_{2}\right)\cdot g^{2}\\ {\hat{m}}_{T}\leftarrow m_{T}/\left(1-\beta_{1}^{T}\right)\\ {\hat{v}}_{T}\leftarrow v_{T}/\left(1-\beta_{2}^{T}\right)\\ \psi_{T}\leftarrow \psi_{T-1}-\alpha \cdot {\hat{m}}_{T}/(\sqrt{{\hat{v}}_{T}}+\varepsilon ). \end{array}$$

In Equation (18), $m_{T}$ is the updated biased first moment estimate, ${\hat{m}}_{T}$ is the bias-corrected first moment estimate, $v_{T}$ is the updated biased second moment estimate, and ${\hat{v}}_{T}$ is the bias-corrected second moment estimate. The subscript T (time step) represents the parameter value for the iterations. After the termination condition has been satisfied, the optimizer will return the parameter values.

#### 3.2.2. Backward Propagation Formula

Rumelhar et al. [25] introduced the back-propagation rule. This method involves calculating the gradient of the output related to the input of each network layer. According to the chain rule, the gradient of the loss value related to the parameter is the product of the calculated gradients of all of the layers. 

The back-propagation method can simplify the gradient calculation of a complex function model, and the intermediate variables are also used to calculate the gradient values, enabling better control of the entire iteration and convergence process.

The CSNF gradient calculation process is also based on the principle of back propagation. To simplify the expression, the output of the three transformation layers is defined as:

(19) $$X_{i}=\left(\begin{array}{c} V_{i}\\ U_{i} \end{array}\right),\;i=1,2,3.$$

The amplitude and DC offset parameters have the same gradient calculation formula:

(20) $$\frac{\partial L}{\partial P}=\frac{\partial L}{\partial Y}\cdot \frac{\partial Y}{\partial X_{3}}\cdot \frac{\partial X_{3}}{\partial X_{2}}\cdot \frac{\partial X_{2}}{\partial X_{1}}\cdot \frac{\partial X_{1}}{\partial P},\;P=a_{1},a_{2},b_{1},b_{2}.$$

The output Y is a scalar, and the input X is a column vector. The gradient of the output with respect to the input for each layer can be calculated according to following formulas:

(21) $$\begin{array}{c} \frac{\partial L}{\partial Y}=-2\cdot \left(1-Y\right),\;\frac{\partial Y}{\partial X_{3}}=\frac{X_{3}^{T}}{Y}\\ \frac{\partial X_{3}}{\partial X_{2}}=\left(\begin{array}{cc} 1/l & 0\\ 0 & 1/s \end{array}\right),\;\frac{\partial X_{2}}{\partial X_{1}}=\frac{\sqrt{2}}{2}(\begin{array}{cc} 1 & -1\\ 1 & 1 \end{array}). \end{array}$$

The expressions of the first layer output related to the parameter are:

(22) $$\frac{\partial X_{1}}{\partial a_{1}}=\left(\begin{array}{c} 0\\ -\frac{u-b_{1}}{a_{1}^{2}} \end{array}\right),\;\frac{\partial X_{1}}{\partial a_{2}}=\left(\begin{array}{c} -\frac{u-b_{2}}{a_{2}^{2}}\\ 0 \end{array}\right),\;\frac{\partial X_{1}}{\partial b_{1}}=\left(\begin{array}{c} 0\\ -\frac{1}{a_{1}} \end{array}\right),\;\frac{\partial X_{1}}{\partial b_{2}}=\left(\begin{array}{c} -\frac{1}{a_{2}}\\ 0 \end{array}\right).$$

All of the intermediate quantities required for the gradient calculations are obtained in the forward-propagation process.

Regarding the coefficient layer, the intermediate output is defined as:

(23) $$t_{1}=\left(\begin{array}{c} \frac{1}{1+t}\\ \frac{1}{1-t} \end{array}\right),\;t_{2}=\sqrt{1+t^{2}}\times t_{1}=\left(\begin{array}{c} \frac{1}{l}\\ \frac{1}{s} \end{array}\right).$$

An operation symbol ⊗ for two vectors is defined. This operation generates a new vector. The elements of the new vector are equal to the products of the two corresponding elements of the previous vectors. Thus $X_{3}=t_{2}⊗X_{2}$.

The parameter related to β is t. Its gradient calculation formula is:

(24) $$\frac{\partial L}{\partial t}=\frac{\partial L}{\partial Y}\cdot \frac{\partial Y}{\partial X_{3}}\cdot \frac{\partial X_{3}}{\partial t_{2}}\cdot \frac{\partial t_{2}}{\partial t}.$$

The gradient of the output with respect to the input for each layer can be calculated by using

(25) $$\begin{array}{c} \frac{\partial L}{\partial Y}=-2\cdot \left(1-Y\right),\;\frac{\partial Y}{\partial X_{3}}=\frac{X_{3}^{T}}{Y}\\ \frac{\partial X_{3}}{\partial t}=\left(\begin{array}{cc} V_{2} & 0\\ 0 & U_{2} \end{array}\right),\;\frac{\partial X_{2}}{\partial X_{1}}=\frac{1}{\sqrt{1+t^{2}}}(\begin{array}{c} \frac{t-1}{{\left(1+t\right)}^{2}}\\ \frac{t+1}{{\left(1-t\right)}^{2}} \end{array}). \end{array}$$

After obtaining the convergence value of t, β can be obtained by using the formula β=2arctan(t).

### 3.3. Summary 

In this section, the design of the CSNF model was presented, where the input and output of the network do not provide explicit angle information, the network input consists of the measured signals, and the loss value of the output is set according to the structure. Then, the use of the back-propagation method to derive gradient formulas for the loss value with respect to the parameters to be identified was described. Finally, the parameter update process based on the Adam optimization algorithm was discussed.

## 4. Simulation and Experiment

### 4.1. Simulation Without Noise 

**Figure 7:**
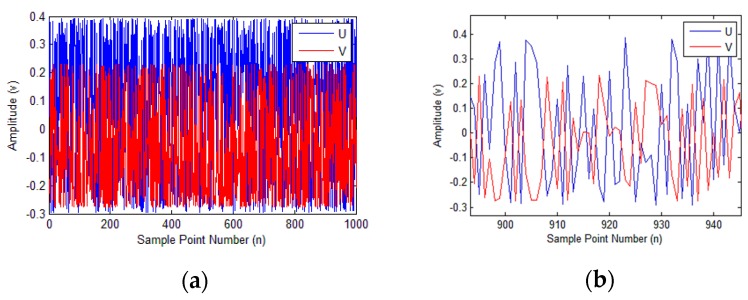

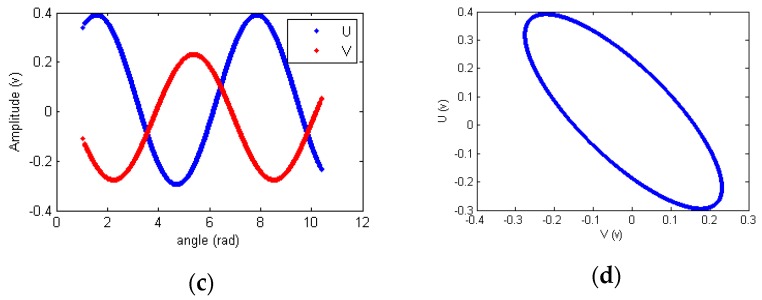
Signals for the simulation. (**a**) Signal amplitude for each sample point; (**b**) details of (a) showing the discontinuity of the signal; (**c**) signal amplitude as a function of input angle; (**d**) Lissajous figure for the simulation signals.

To verify the feasibility of the identification algorithm, a simulation experiment was conducted using a software platform. The amplitudes, DC offsets, and phase shift were set based on the signal model of the sensor. An unordered angle signal was then generated, and the two sets of output values were saved, which could simulate the discontinuity of the input angle. In the simulation, *a*_1_ and *a*_2_ were set to 0.3428 and 0.2537, respectively; *b*_1_ and *b*_2_ were set to 0.0485 and −0.0224, respectively; and *β* was set to 0.9032. The signals for the simulation are depicted in Figure 7.

Signal flow network, back-propagation gradient calculation, and Adam optimization methods were followed for the algorithm design and simulation experiment. In the training data initialization process, the maximum absolute values of the two output signals were used as the initial amplitudes, the initial DC offsets and phase shift were set to 0. Using a small batch of gradient descent can both ensure accuracy and improve the training speed [23]. Batch training was used to update the weights, using a batch size of 100.

During the parameter updating process, the learning rate α was set to 0.01 to speed up the convergence rate, while all of the other parameters were in accordance with the recommended values in [24].

**Figure 8:**
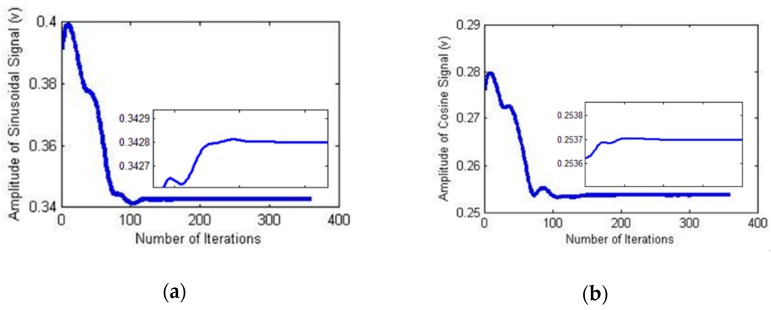
Estimation process. (**a**) Amplitude of the sine signal; (**b**) amplitude of the cosine signal.

The parameter and loss function values were collected during the network training process to analyze their convergence. The amplitude variation during the CSNF training process is depicted in Figure 8.

**Figure 9:**
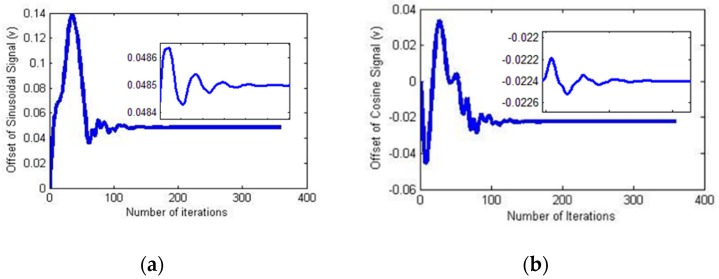
Estimation process. (**a**) DC offset of the sine signal; (**b**) DC offset of the cosine signal.

The variation of the DC offset during the CSNF training process is illustrated in Figure 9.

**Figure 10:**
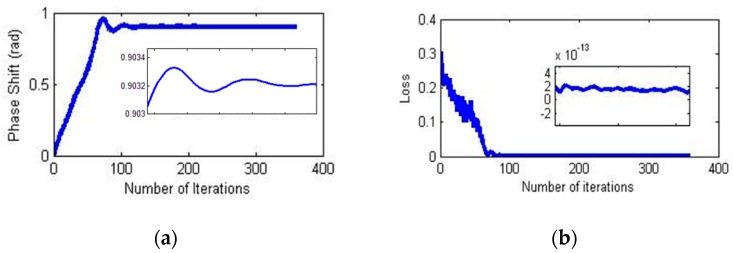
Estimation process. (**a**) Phase shift between two signals; (**b**) loss value during training.

The changes in the phase shift and loss values during the training process are shown in Figure 10.

**Figure 11:**
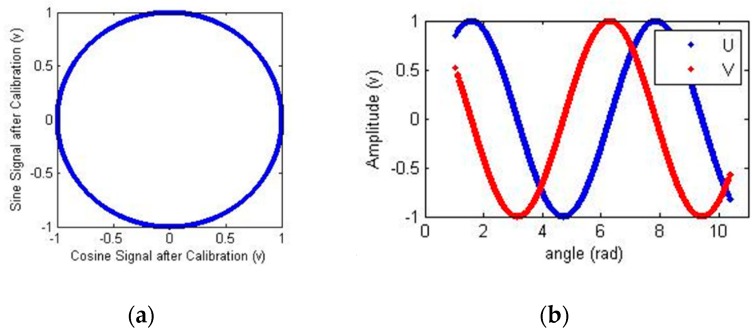
Calibrated signals. (**a**) Calibrated Lissajous figure; (**b**) calibrated sine and cosine signals.

According to the training results, the calibrated signals and Lissajous figure were obtained and are presented in Figure 11.

The parameter information is summarized in Table 1. The results show that the CSNF method could provide high-precision parameter identification results, confirming the theoretical feasibility and effectiveness of the CSNF method.

**Figure 12:**
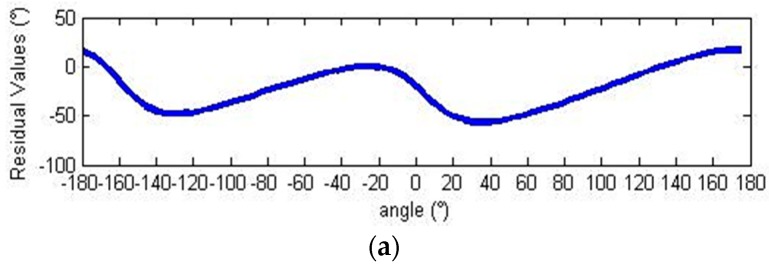

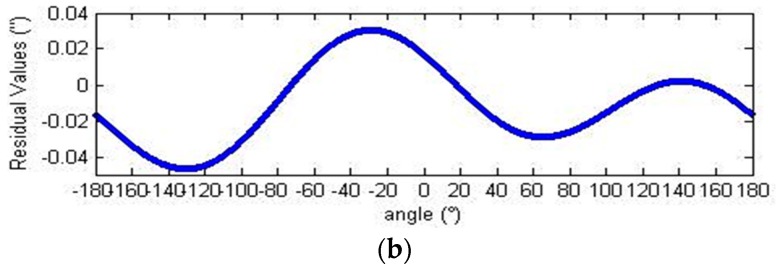
Demodulation errors. (**a**) Demodulation error before calibration; (**b**) demodulation error after calibration.

The residual values of the demodulation angle are depicted in Figure 12.

Before calibration, the peak-to-peak angle error was 73.72°. After calibration, it was reduced to about 0.09 arc sec, corresponding to an error of 0.00003%. 

### 4.2. Simulation with Harmonic Components 

The next simulation verified the feasibility of the scheme. Specifically, the calibration effects with harmonic components present were verified for practical applications.

**Figure 13:**
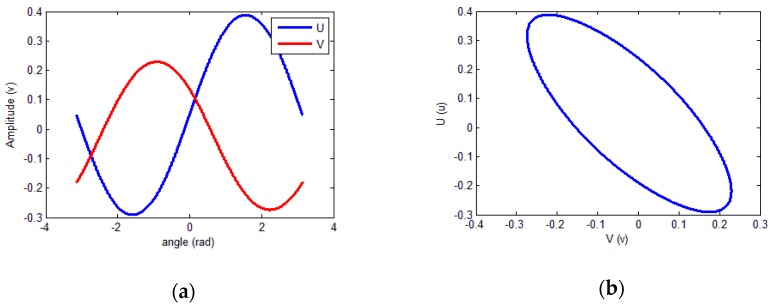
Signals for the simulation with harmonic components. (**a**) Amplitude versus input angle; (**b**) Lissajous figure for the simulation signals.

Harmonic components are common in sensor output signals. To verify the effects of the harmonic components on the compensation, triple frequency components were added to the simulation model. The amplitude of the triple frequency components was set to 0.01 of the fundamental frequency. The signals for the simulation are presented in Figure 13.

**Figure 14:**
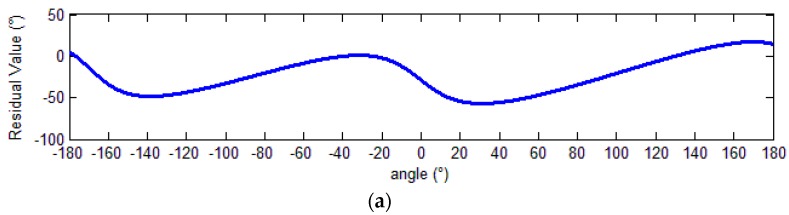

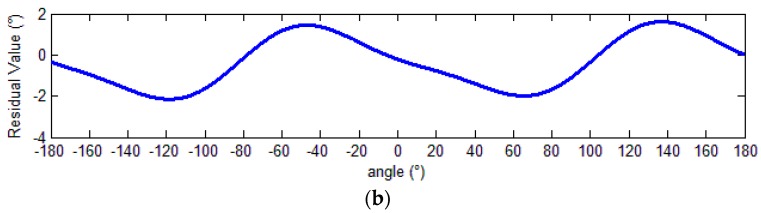
Demodulation errors. (**a**) Demodulation error before calibration; (**b**) demodulation error after calibration.

The CSFN method was used for parameter identification and signal calibration. The angle demodulation errors are depicted in Figure 14.

With harmonic components, the peak-to-peak angle error was 74.17° before calibration and was reduced to 3.77°, or 5.08%, after compensation. Thus, the compensation effect became worse than it was without harmonic components.

### 4.3. Experimental Results

To verify the effectiveness of the proposed technique in practical applications, experiments were conducted using a CAPS and a turntable.

**Figure 15:**
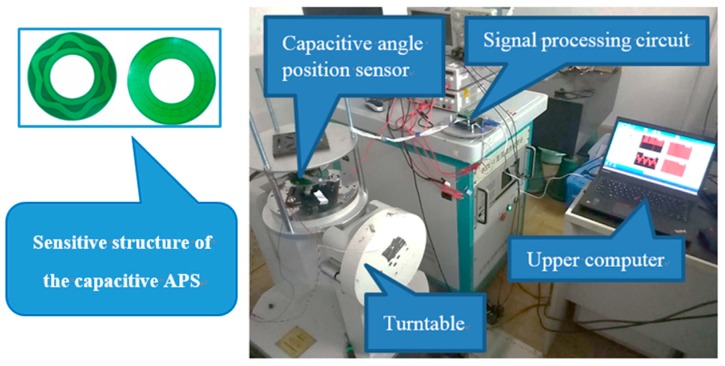
Experimental equipment, including a turntable, a CAPS, a signal processing circuit, and an upper computer.

Figure 15 illustrates the experimental equipment used for verification.

In the experiment, a CAPS [6] was mounted on a turntable, which rotated at 0.5°/s. During the rotation process, the signal processing circuit processed the signals and sent the data to the upper computer at a sampling frequency of 250 Hz. The exact values of the rotation angles were obtained through the turntable, and parameter identification was performed offline.

**Figure 16:**
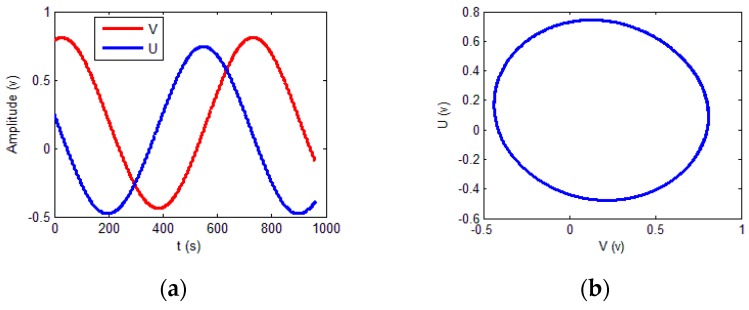
Signals for experiment. (**a**) Sinusoidal and cosine signals; (**b**) Lissajous figure for the output signals.

The output signals of the sensor are presented in Figure 16.

**Figure 17:**
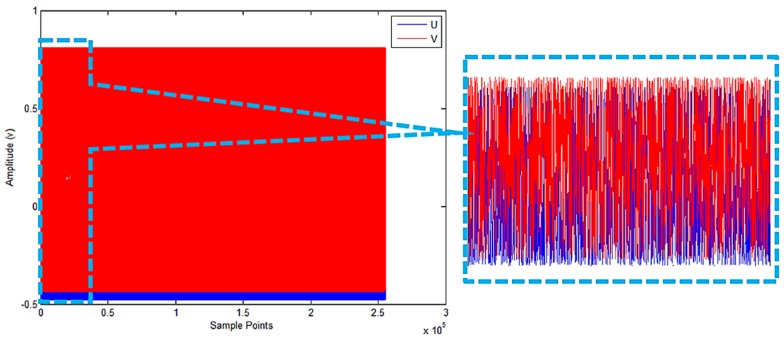
Shuffled experimental signal data.

The previous experimental data were continuous. To verify that the proposed scheme can be used for non-continuous data, the data were randomly shuffled. The shuffled signals are shown in Figure 17. 

The presence of noise is not conducive to stable gradient changes. Since larger batches can make the gradient change process smoother, the batch size was set to 1000. The parameter and loss function values were collected to analyze the convergence of the parameters and loss functions.

**Figure 18:**
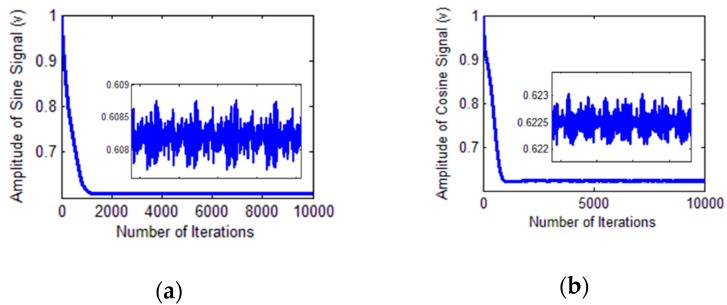
Identification process. (**a**) Amplitude of the sine signal; (**b**) amplitude of the cosine signal.

The amplitude identification process is illustrated in Figure 18.

**Figure 19:**
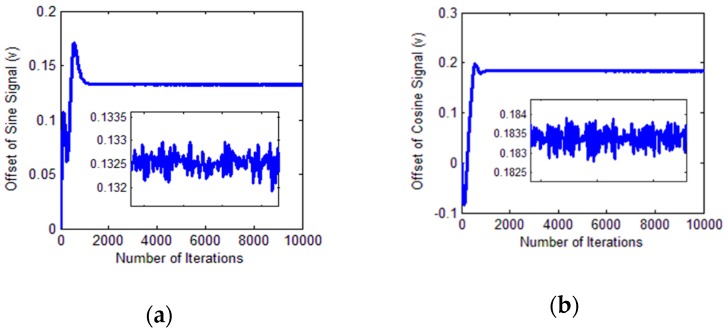
Identification process. (**a**) DC offset of the sine signal; (**b**) DC offset of the cosine signal.

The DC offset identification process is shown in Figure 19.

**Figure 20:**
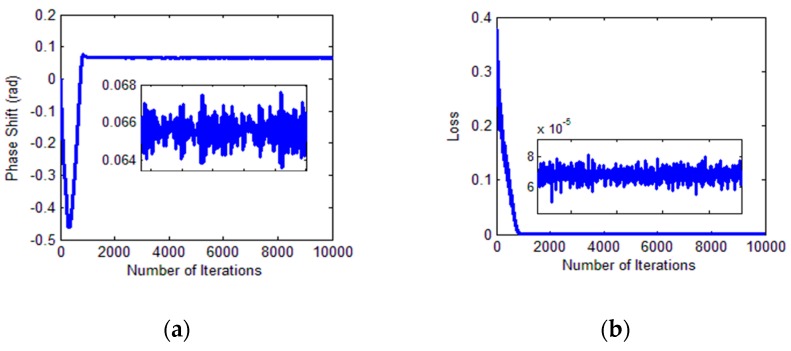
Identification process. (**a**) Phase shift of two signals; (**b**) loss value during training.

The changes in phase shift and loss during training process are shown in Figure 20.

**Figure 21:**
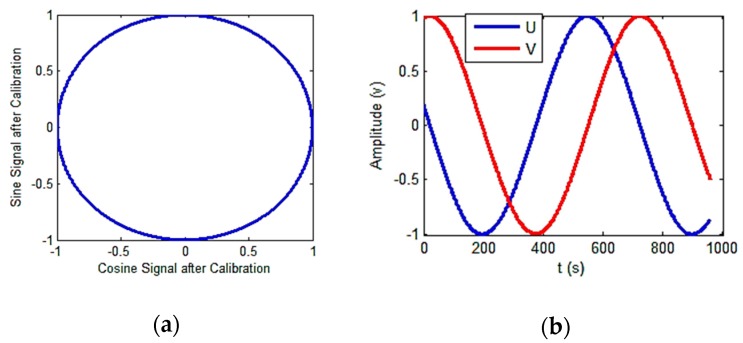
Calibrated signals. (**a**) Calibrated Lissajous figure; (**b**) calibrated sine and cosine signals.

According to the training results, the calibrated output signal and its Lissajous figure were obtained and are presented in Figure 21.

The parameter identification results are summarized in Table 2.

**Figure 22:**
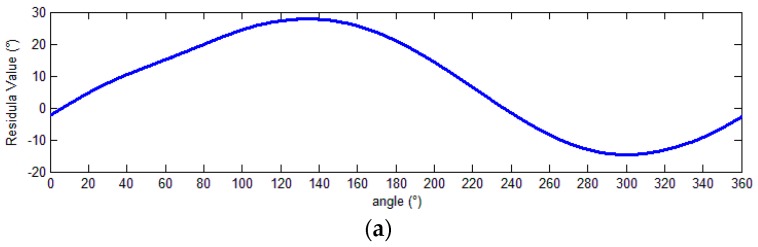

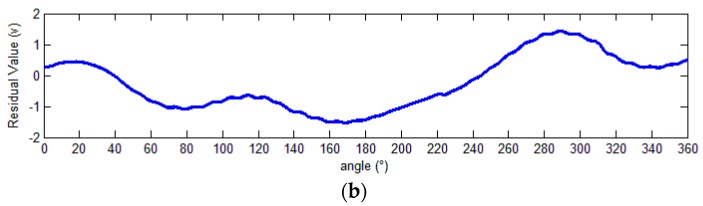
Demodulation errors. (**a**) Demodulation error before calibration; (**b**) demodulation error after calibration.

The residual values of the demodulation angle are shown in Figure 22.

Before calibration, the peak-to-peak angle error 42.57°. After calibration, it was reduced 2.97°, representing an error of only 6.98%. The experimental results demonstrate that this scheme can effectively calibrate output signals and improve the angle resolution accuracy.

**Figure 23:**
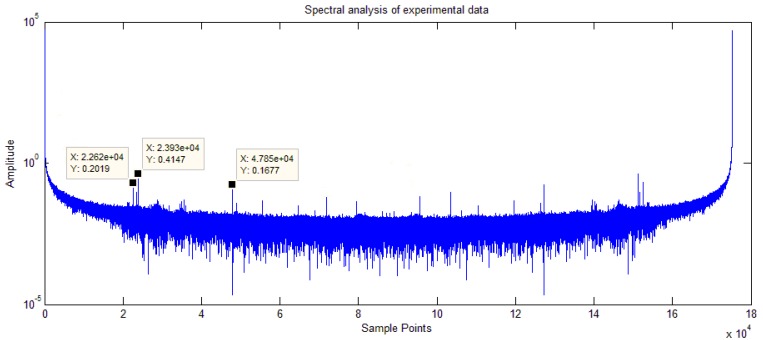
Spectrum analysis of experimental data.

The experimental results are similar to the simulation results obtained with harmonic components. For further analysis, a Fourier transform of the experimental data with the signal model $U=a_{1}\cdot \sin\theta +b_{1}$ was performed. The results are presented in Figure 23.

The spectrum analysis chart shows that the output signal contained high frequency components, indicating that the actual signal differed from the signal model in the literature [6,17]. Considering the existence of harmonic components, the experimental results are consistent with the simulation results. 

The experimental results show that the demodulation error was reduced to only 6.98%. To improve the compensation accuracy further, the effects of the harmonic components must be analyzed. The suppression of harmonic components requires more complete and complex models, and future work will focus on using the CSFN method to address this problem.

## 5. Discussion

Self-calibration to improve the accuracy of an APS when the input signal is unknown is commonly performed and necessary in practical applications. This article proposes the CSFN method as a means of identifying the parameter values of the signal model. Through iterative learning, this approach can provide accurate model parameters and output calibrated sensor signals. The simulation and experiment results verify the validity of the proposed method. For the harmonic components of the output signals, future research will focus on using this method to address more complete and complex signal models to calibrate the harmonic components and reduce the demodulation error.

## 6. Conclusions

This article proposes a self-calibration scheme for angle sensor signals. The simulation results demonstrate that this method can yield high-accuracy identification results. Analyses of the effects of the harmonic components on the compensation were also presented. The obtained experimental and simulation results are basically consistent and verify the feasibility of the proposed method. To improve the calibration effect further, future work will focus on identification in the presence of harmonic components and obtaining more accurate angle signals.

## Figures and Tables

**Table 1:** Parameter estimation results obtained from the simulation.

Parameter	Preset Value	Identification Value	Relative Error
$a_{1}$ (V)	0.3428	0.3428	$2.53\times 10^{-7}$
$a_{2}$ (V)	0.2537	0.2537	$2.12\times 10^{-7}$
$b_{1}$ (V)	0.0485	0.0485	$-5.77\times 10^{-7}$
$b_{2}\;$(V)	−0.0224	−0.0224	$-7.14\times 10^{-7}$
β (rad)	0.9032	0.9032	$1.70\times 10^{-7}$

**Table 2:** Parameter estimation results from the experiment.

Parameter	Identification Value
$a_{1}$ (V)	0.6083
$a_{2}$ (V)	0.6223
$b_{1}$ (V)	0.1324
$b_{2}$ (V)	0.1830
β (rad)	0.0657
